# Response to Liu et al.

**DOI:** 10.14309/ctg.0000000000000096

**Published:** 2019-11-01

**Authors:** Mark O. Goodarzi, David C. Whitcomb

**Affiliations:** 1 Division of Endocrinology, Diabetes, and Metabolism, Department of Medicine, Cedars-Sinai Medical Center, Los Angeles, California, USA; 2 Division of Gastroenterology, Hepatology, and Nutrition, Department of Medicine, University of Pittsburgh and UPMC Medical Center, Pittsburgh, Pennsylvania, USA; 3 Department of Human Genetics, Graduate School of Public Health, University of Pittsburgh, Pittsburgh, Pennsylvania, USA; 4 Department of Cell Biology and Molecular Physiology, University of Pittsburgh, Pittsburgh, Pennsylvania, USA.

We thank Liu et al. for their letter and hope that the response will answer their concerns and will also be informative to others who are confused about these issues. Three points will be addressed.

## RELATIONSHIP BETWEEN RECURRENT ACUTE PANCREATITIS AND CHRONIC PANCREATITIS

The clinicopathologic definitions of recurrent acute pancreatitis (RAP) and chronic pancreatitis (CP) are based on distinguishing clinical features of complex syndromes (e.g., >1 episode of AP for the diagnosis of RAP and morphologic features of fibrosis for the diagnosis of CP) with no reference to etiology. In the 1960s, attempts were made to distinguish RAP from CP ((1), see also Etemad and Whitcomb (2)), but the discovery that gain-of-function mutations p.R122H or p.N29I in the cationic trypsinogen gene (*PRSS1*) cause AP and RAP (primary phenotype), with a subset later developing CP, clarified the progressive relationship between these clinical syndromes (3).

The etiology-based, progressive relationship between RAP and CP is specifically defined in the Mechanistic Definition of CP (4), a new definition that has been accepted by the major pancreatic societies (5). Multiple studies, including the meta-analysis (6) noted by Liu et al., confirm the relationship between RAP and CP, but they do not infer that all RAP becomes CP nor that CP requires RAP. Note also that the diagnosis of RAP does not preclude the diagnosis of CP or *vice versa*, and they can occur in the same person at the same time.

The evidence that Liu et al. use to argue against including RAP with CP in our analysis is based on an article by Cavestro et al. (7). However, this article is now a decade out of date, grossly underpowered (RAP n = 64; CP n = 142), and only looked at 1 variant in *MCP-1*, 1 in *SPINK1*, and 33 cystic fibrosis-causing variants in *CFTR*. Their limited data suggested that genetic variants cause RAP but not CP. More recent studies that are larger and/or that have deeper genotyping demonstrate that RAP and CP have common etiologies and that time and persistent injury or inflammation, or additional risk factors that are present within a subset of RAP patients, cause progression to CP (8–12). Finally, the etiology of diabetes mellitus (DM) is linked to the pancreatic beta cell, not the acinar or duct cells that contribute to the etiology of RAP and CP. The fact that even one episode of AP clearly increases risk of DM (13) suggests that in some patients, susceptibility to DM is clearly linked to exocrine pancreatic injury and inflammation—which takes the forms of AP, RAP, and CP.

## NORTH AMERICAN PANCREATITIS STUDY II

We are happy to clarify the study cohorts used by Goodarzi et al. (14) related to the North American Pancreatitis Study II (NAPS2) samples. Liu et al. inferred bias in sample selection of this study based on counting sample from 2 previous reports using NAPS2 samples (9,15). However, a simple reading of the methods papers reveals that the genome-wide association study included only cases (both RAP and CP) and controls of European ancestry and additional non-NAPS2 samples from England, Germany, and the United States. This is appropriate for detecting genetic variants associated with pancreatitis. However, the non-NAPS2 samples were not phenotyped for DM and could not be used in more detailed analyses.

The second article referenced by Liu et al. focused on patients with non-European ancestry (i.e., African Americans) (15) and did not include the genotyping results, since the initial genome-wide association study chip was designed for patients of European ancestry (9). Furthermore, there was continued patient recruitment to the Pittsburgh site, further increasing the overall cohort size.

The sample selected for the study by Goodarzi et al. (14) includes only NAPS2 subjects since they had detailed information of the presence of absence of DM, the age of DM and AP, RAP and CP onset, and medications. All NAPS2 subjects with complete case report forms, and in which genotyping on all SNPs were successfully genotyped, were indeed included.

Liu et al. also raised concerns about a selection bias for not having follow-up data on the patients for at least 2–5 years to exclude pancreatic cancer, referencing Kirkegard et al. (16). We are unclear as to the relevance of this criticism since (i) the study by Kirkegard focused on AP, not CP, (ii) the follow-up time requirement was to insure that AP was not caused by pancreatic cancer, (iii) the study used an administrative data set, so that unlike the NAPS2 patients, the cases were not phenotyped by an expert physician with access to imaging studies and laboratory values within a clinical context, and (iv) even after 5 years of follow-up, the incidence of pancreatitis cancer was less than 1%. Although it is also possible that patients with CP may develop pancreatic cancer and that a subset of these patients may have glucose intolerance or DM (17), the mechanism is likely paraneoplastic and would bias the study against type 2 DM (T2DM) genetics (18). Thus, we do not believe the study by Goodarzi was biased by contamination by patients with pancreatic cancer.

## HETEROGENEITY OF DIABETES ETIOLOGY

Liu et al. suggest that we did not clearly highlight the heterogeneity within the group with CP and diabetes (CP-DM). On the contrary, we discussed at length the likelihood that the CP-DM group included people with diabetes caused by CP and people with classic T2DM. We even acknowledged that both etiologies may contribute to diabetes in some individuals. We explicitly stated that such heterogeneity could have reduced the ability for the genetic risk score (GRS) to distinguish the CP-DM group from the T2DM group. The potential heterogeneity is why we conducted additional analyses attempting to enrich the CP-DM group with pancreatogenic diabetes or deplete the group of T2DM. Regarding the latter, Liu et al. suggest we should have simultaneously excluded patients with multiple T2DM-related features in subanalysis. We had examined the strategy of stratifying on multiple features but found that it resulted in CP-DM subgroups of small size and generally did not add insight beyond considering single features. As an example, we provide the analysis requested by Liu et al. A CP-DM subgroup of 63 patients had diabetes occurring after pancreatitis, no family history of diabetes, and was not obese or overweight. Their mean GRS was 66.21, which was essentially identical to the mean GRS of 66.42 in the 423 patients with T2DM (*P* = 0.93).

Thus, we stand by the study design and the conclusions and hope that this additional explanation of our previous articles and review of the genetics literature will bring clarity to this changing field.

## CONFLICTS OF INTEREST

**Guarantor of the article:** Mark O. Goodarzi, MD, PhD.

**Specific author contributions:** M.O.G., D.C.W.: drafting of manuscript.

**Financial support:** This research was partly supported by NIH grants R01-DK061451 (DCW), U01-DK108314 (MOG), U01-DK108306 (DCW), P30-DK063491 (MOG), and the Eris M. Field Chair in Diabetes Research (MOG). The study sponsors had no role in the study design, collection, analysis or interpretation of data, in the writing of the report, or in the decision to submit the report for publication.

**Potential competing interests:** D.C.W. is a consultant for AbbVie, Regeneron, and Ariel Precision Medicine and has equity in Ariel Precision Medicine. M.O.G. has no conflicts to disclose.
